# Encapsulated stem cell–derived β cells exert glucose control in patients with type 1 diabetes

**DOI:** 10.1038/s41587-023-02055-5

**Published:** 2023-11-27

**Authors:** Bart Keymeulen, Kaat De Groot, Daniel Jacobs-Tulleneers-Thevissen, David M. Thompson, Melena D. Bellin, Evert J. Kroon, Mark Daniels, Richard Wang, Manasi Jaiman, Timothy J. Kieffer, Howard L. Foyt, Daniel Pipeleers

**Affiliations:** 1 Diabetes Research Center, Vrije Universiteit Brussel and Universitair Ziekenhuis Brussel, Brussels, Belgium; 2 Division of Endocrinology, Department of Medicine, University of British Columbia, Vancouver, British Columbia Canada; 3 Department of Pediatrics and Department of Surgery, University of Minnesota Medical Center, Minneapolis, MN USA; 4 ViaCyte Inc., San Diego, CA USA; 5 Department of Cellular and Physiological Sciences and Department of Surgery, University of British Columbia, Life Sciences Institute, Vancouver, British Columbia Canada

**Keywords:** Stem-cell differentiation, Type 1 diabetes

## Abstract

Clinical studies on the treatment of type 1 diabetes with device-encapsulated pancreatic precursor cells derived from human embryonic stem cells found that insulin output was insufficient for clinical benefit. We are conducting a phase 1/2, open-label, multicenter trial aimed at optimizing cell engraftment (ClinicalTrials.gov identifier: NCT03163511). Here we report interim, 1-year outcomes in one study group that received 2–3-fold higher cell doses in devices with an optimized membrane perforation pattern. β cell function was measured by meal-stimulated plasma C-peptide levels at 3-month intervals, and the effect on glucose control was assessed by continuous glucose monitoring (CGM) and insulin dosing. Of 10 patients with undetectable baseline C-peptide, three achieved levels ≥0.1 nmol l^−1^ from month 6 onwards that correlated with improved CGM measures and reduced insulin dosing, indicating a glucose-controlling effect. The patient with the highest C-peptide (0.23 nmol l^−1^) increased CGM time-in-range from 55% to 85% at month 12; β cell mass in sentinel devices in this patient at month 6 was 4% of the initial cell mass, indicating directions for improving efficacy.

## Abstract (web summary)

β cells derived from stem cells improve blood glucose control in patients with diabetes.

## Main

In type 1 diabetes (T1D), the pancreas is depleted of β cells and, hence, of cell-regulated provision of insulin according to metabolic needs. Exogenous insulin can substitute for the loss of the insulin reserve but not for fine-tuned cellular control of its synthesis and release. Insulin administration reduces hyperglycemic episodes in most patients, thus avoiding or delaying progression toward chronic complications, but also carries a risk of life-threatening hypoglycemic events and adds to the burden of living with a chronic disease. The potential of β cell replacement as a cure for T1D was shown by the outcome of intrahepatic transplants of islets prepared from human donor pancreases^1–3^. This form of cell therapy resolves the hypoglycemic events in most recipients and, thereby, substantially increases their quality of life^3,4^. Moreover, the procedure can virtually normalize glucose control and eliminate the need for exogenous insulin therapy for more than 50% of patients over 5 years^5^. Implementation is limited by the shortage of donor cells and by the need for continuous immunosuppressive treatment to prevent graft rejection, which introduces a risk of life-threatening complications.

In principle, both the shortage of donor cells and the requirement for immunosuppression could be addressed by using fully encapsulated β cells derived from human pluripotent stem cells (hPSCs). Methods have been developed for the directed differentiation of hPSCs to pancreatic endoderm (PE)^6^ and subsequently to β cell–containing preparations^7–9^. Transplantation of cells at both the pancreatic endoderm and β cell stages of differentiation can generate functional β cell implants in immune-compromised rodents^7,8,10^. Cell encapsulation has been explored as a means of providing a barrier to protect the recipient against invading donor cells and the donor cells against infiltrating immune cells^11–14^. Moreover, such a device can physically constrain any off-target cells^15^ and, when placed in an appropriate site, offer the additional advantage of being accessible for environmental adaptations and being retrievable for analysis or for safety.

The challenge of keeping transplanted β cells alive in a sealed encapsulation device motivated the development of perforated or ‘open’ devices that allow ingrowth of capillaries to improve cell survival. The attainment of durable glycemic control with open device–contained hPSC-derived pancreatic endoderm cells (PECs) implanted in the subcutis of immune-compromised mice and rats led to the manufacture of a combination product (PEC-Direct) intended for phase 1/2 studies in patients with T1D^16^. This product consists of devices with perforated membranes, which are loaded with pancreatic endoderm cells (PEC-01) obtained by differentiation of the CyT49 human embryonic stem cell line^6,16^. The perforations allow ingrowth of capillaries but also of host immune cells, so immunosuppression, adapted from clinical islet allo-transplantation settings, is used. Although the membranes do not provide an immune-protecting barrier, the encapsulation device is intended to contain the PEC-01 cells and their progeny at the implant site.

Two recent studies tested the safety of PEC-Direct in immunosuppressed patients with T1D and reported the first clinical evidence for formation of functional β cells in the implants^17,18^. Insulin-positive cells were identified in retrieved implants, and plasma C-peptide was detected after meal stimulation. Plasma C-peptide levels are an in vivo marker for the presence and size of a functional β cell mass and its glucose responsiveness; they have been correlated with the ability of a β cell mass to exert glucose control^19^. However, although a small increase in C-peptide was detected^17,18^, it did not reach levels ≥0.1 nmol l^−1^, which is considered the threshold for metabolic significance^19,20^, and, accordingly, no improvement in glycemic control could be attributed to the implants^18^. Therefore, subsequent study groups were designed to examine whether greater efficacy could be achieved with changes in device configuration and/or implant strategy (Methods, ‘Global description of clinical trial’ subsection).

Here we describe 1-year follow-up in a study group of 10 patients with T1D who (1) received twofold to threefold more devices than in the previous studies and (2) received devices with a membrane perforation density and pattern^21^ that was previously associated with higher cell survival in devices and with higher plasma C-peptide induction^17,18^. Four of 10 recipients achieved the primary endpoint of detectable plasma C-peptide at month 6. Three of these four recipients achieved C-peptide ≥0.1 nmol l^−1^ and exhibited an improvement in glucose control at month 6 and month 9 as indicated by continuous glucose monitoring (CGM) measures together with a decrease in exogenous insulin dosing. Quantitative analysis of the cellular composition in devices retrieved from the patient with the best outcome (case 1) indicated that the β cell mass achieved at month 6 was 4% of the initial cell mass, and its proportion was low (3% of all cells)—fivefold lower than the α cell mass (16% of all cells).

## Results

### Overview of trial design

The trial examined efficacy of a subcutaneous implant of PEC-Direct in patients with T1D under immune suppression, which was induced by anti-thymocyte globulin and maintained by mycophenolate mofetil and tacrolimus (Methods and Supplementary Table 1). The implant consisted of eight (cases 1 and 6) or 10 large (dose-finding) and two or three (case 1) small (sentinels for analysis) units, each containing, respectively, ~75 × 10^6^ and ~7 × 10^6^ PEC-01 cells (Methods). At screening, the 10 patients presented a state of β cell depletion as shown by plasma C-peptide levels below the limit of detection (LOD) (0.03 nmol l^−1^) at basal and at minute 90 of a mixed meal tolerance test (MMTT). The primary endpoint was an increase of MMTT-induced C-peptide above the LOD at month 6. The secondary endpoints were a C-peptide level >0.07 nmol l^−1^, improvement of CGM measures and reduced insulin dosing over 1-year follow-up (Methods, ‘Global description of clinical trial’ subsection). The functionality of the implants in improving glucose control was assessed by joint examination of C-peptide levels, CGM data and insulin dosing.

Treatment-emergent adverse events (TEAEs) were recorded. None led to withdrawal from the study, and none corresponded to adverse events of special interest (AESIs) as defined in the protocol. The most common TEAE was procedural pain. Two patients reported treatment-emergent serious adverse events (TESAEs), one attributable to the surgical procedure and one to the protocol-specified immunosuppression.

### Meal-stimulated plasma C-peptide in recipients of PEC-Direct

**Fig. 1:**
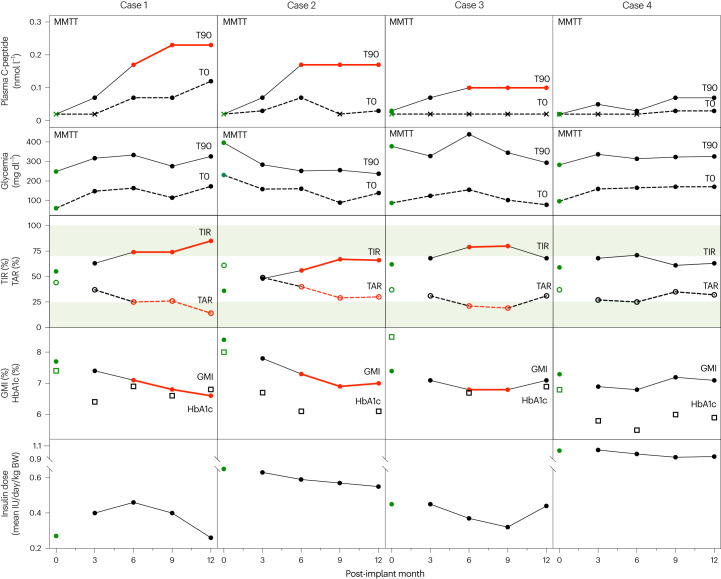
Markers of glucose control in T1D recipients of PEC-Direct implant achieving efficacy endpoints of implant function. Patients who achieved the primary efficacy endpoint for implant function (6-month MMTT-stimulated C-peptide at minute 90 above baseline; Table 1) were examined for associated changes in glucose control markers over 12 months. The first (top) panel presents plasma C-peptide levels at basal (T0, minute 0) and at MMTT-stimulated (T90, minute 90) timepoints at 3-month intervals; levels ≤LOD are indicated by a cross. Cases 1, 2 and 3 achieved the secondary efficacy endpoint for implant function (plasma C-peptide > 0.07 nmol l^−1^) and maintained it until month 12 (red line). Glycemia at these MMTT points (second panel) indicates the glucose stimulation state at minute 90. The third and fourth panels show CGM-derived endpoints of glucose control over 3-month intervals. TAR glycemia (≥180 mg dl^−1^) and TIR glycemia (71–180 mg dl^−1^) have been defined as core endpoints of glucose control with targets (TIR >70% and TAR <25%, shaded areas in the third panel)^24,25^. The three recipients who met the secondary endpoint of implant function (cases 1, 2 and 3) improved CGM measures toward the clinical targets at month 6 and month 9, two of them also at month 12 (red lines), whereas no improvement was observed for case 4, which did not meet this secondary endpoint. The improvement in glucose control of cases 1, 2 and 3 is also expressed by the reduction in the CGM-derived GMI (red lines, fourth panel), which more accurately represents changes in mean glycemia than the HbA1c levels (open squares), which are measured only at 3-month intervals and which are influenced by the immunosuppressive treatment. The fifth panel shows the corresponding insulin dose. It has been averaged over 2–4 weeks before implantation (month 0, green dots) and over 3-month periods thereafter. Immunosuppressive treatment was initiated 1–10 d before implantation. The different pre-implantation conditions, as well as changes in lifestyle after start of the protocol, make the month 3 dose an appropriate reference to evaluate individual changes over time. The data show that the improvement in glucose control endpoints in cases 1, 2 and 3 (red lines) are associated with lowering of insulin dose, which is consistent with the establishment of a metabolically relevant implant function during this period. No relevant change in insulin dose was noted for case 4. BW, body weight.

At post-implant month 3, five of 10 recipients exhibited MMTT-induced C-peptide levels greater than LOD (0.05–0.07 nmol l^−1^) (Table 1). Detectable C-peptide remained present in four of 10 recipients at month 6, meeting the primary efficacy endpoint, and during longer follow-up. Three of these four patients achieved the secondary endpoint of C-peptide levels >0.07 nmol l^−1^ until month 12 (0.10–0.23 nmol l^−1^) (Fig. 1). No correlation was observed between this responder group and characteristics of the patients at time of implantation (Supplementary Table 1).

**Table 1:** MMTT-induced C-peptide release in PEC-Direct recipients

Cases	Plasma C-peptide level (nmol l^−1^) at MMTT minute 90				
	Pre-implant	Post-implant			
	Screening	Mo 3	Mo 6	Mo 9	Mo 12
1	<0.03	0.07	0.17	0.23	0.23
2	<0.03	0.07	0.17	0.17	0.17
3	0.03	0.07	0.10	0.10	0.10
4	0.03	0.05	0.04	0.07	0.07
5	<0.03	0.07	0.03	0.03	0.03
6	<0.03	0.03	<0.03	<0.03	0.03
7	<0.03	<0.03	<0.03	<0.03	NA
8	<0.03	<0.03	<0.03	<0.03	<0.03
9	<0.03	<0.03	<0.03	<0.03	NA
10	<0.03	<0.03	<0.03	<0.03	NA

Data were collected at glycemia >250 mg dl^−1^ (Methods).

Cases are ranked according to C-peptide level at month (Mo) 6.

NA, not available for withdrawn cases.

**Fig. 2:**
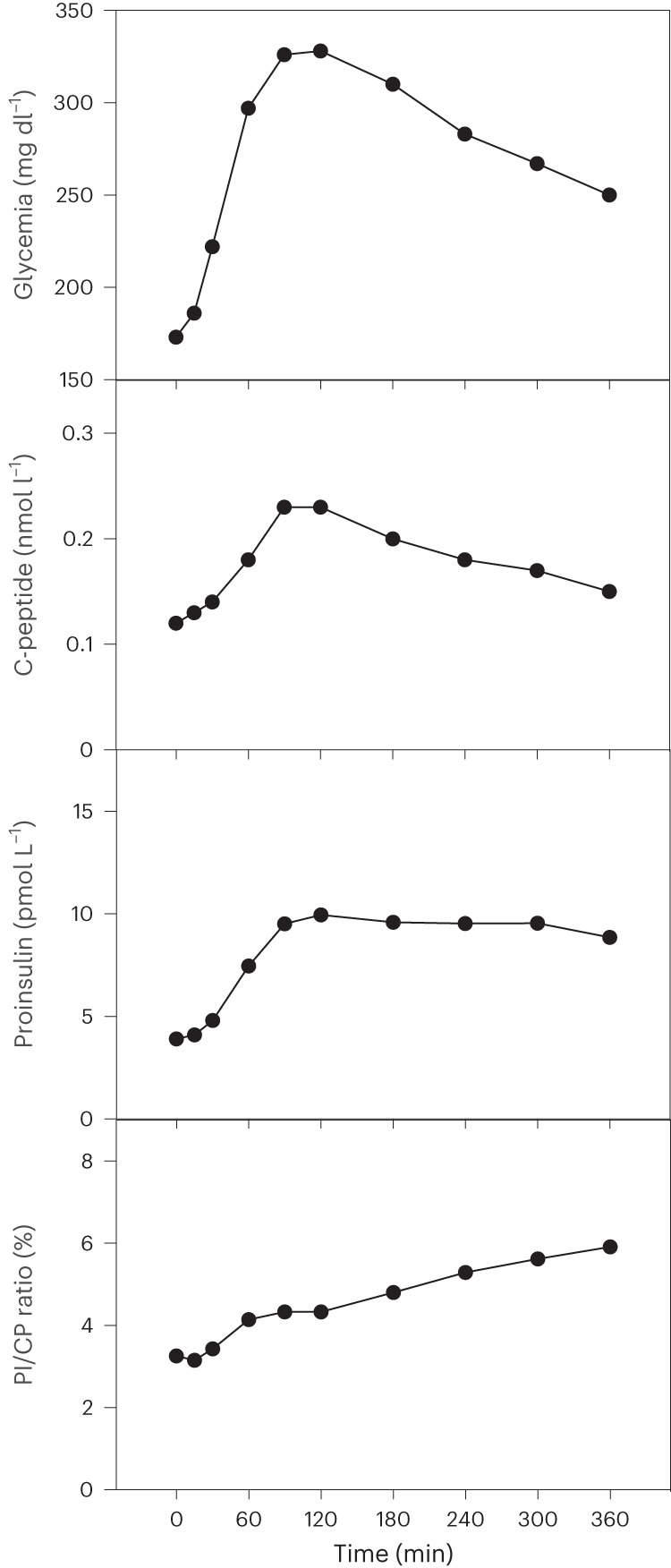
Secretory response of human stem cell–generated β cell mass during MMTT at post-implant month 12. MMTT sampling was prolonged to 360 min in case 1, which maintained β cell function and metabolic control up to month 12. Plasma C-peptide and proinsulin levels increased in parallel to the rise in glycemia, but only proinsulin release maintained its peak level during sustained elevation of glycemia, leading to a progressively increasing molar ratio of proinsulin over C-peptide (PI/CP).

For the case with the best C-peptide secretory response (case 1), MMTT-induced C-peptide increased threefold between month 3 and month 9 and then remained stable. Although the clinical protocol did not plan for proinsulin analysis, we took advantage of the relatively high graft function in this patient to assess proinsulin release as a means of further interrogating the functional state of the stem cell–derived β cells. At month 12, β cell secretory activity was insufficient to correct the hyperglycemic state during a 360-min MMTT (Fig. 2). The β cell mass remained activated during this prolonged hyperglycemia as indicated by the sustained elevation of plasma C-peptide and proinsulin levels; however, the progressive decrease in C-peptide marks a shortage in cellular reserves of processed hormone, as occurs during sustained activation of a β cell mass of insufficient size. An elevated proinsulin/insulin molar ratio was previously shown to mark glucose-activated human β cells with low cellular hormone reserves^22^.

### Improved glucose control in recipients with β cell function

The four patients with induced β cell function, as expressed by the primary efficacy endpoint, were evaluated for their glucose control markers over the 12-month period using recommended glucose monitoring measures obtained during CGM^23,24^ (Supplementary Table 2). Of these measures, time-in-range (TIR; glycemia 71–180 mg dl^−1^), time-above-range (TAR; glycemia >180 mg dl^−1^) and time-below-range (TBR; glycemia ≤70 mg dl^−1^) represent core values for evaluating glucose control. The glucose management indicator (GMI), a secondary endpoint, expresses mean glycemia as a CGM substitute for HbA1c values that are measured only at 3-month intervals^25,26^. The three patients (cases 1–3) who met the primary and secondary endpoints for implant function, with C-peptide response ≥0.1 nmol l^−1^, exhibited an increase in TIR and a decrease in TAR, reaching consensus targets for glucose control (Fig. 1)^25^; TBR remained low (≤4%) without severe hypoglycemic events. This improvement in glucose control measures was persistent from month 6 to month 12, as were the elevated C-peptide levels. It was associated with an improvement in the GMI (Fig. 1). No improvement in these glucose control parameters was observed in case 4, which did not meet the secondary efficacy endpoint for implant function (Fig. 1 and Supplementary Tables 2–4).

In cases 1–3, the period with C-peptide response ≥0.1 nmol l^−1^ (month 6 to month 12) and improved glucose control was associated with a reduction in exogenous insulin dose (Fig. 1). For case 1, daily insulin doses decreased by as much as 44% between month 6 and month 12 and with better glucose control. This patient had required a higher insulin dosing during the first 3 months to adjust for increased body weight (+12%) and twofold higher carbohydrate intake (Supplementary Table 2). For case 2, insulin dose between month 6 and month 12 was 15% lower than pre-implant or during the first 3 months while achieving better glucose control (Fig. 1 and Supplementary Table 2). For case 3, insulin dose was 29% reduced at month 9 with better glucose control, but this effect was not maintained through month 12 (Fig. 1 and Supplementary Table 2). Liraglutide treatment had been started in this patient shortly before month 3 after a site decision to treat the weight gain since inclusion. The introduction of a GLP-1 receptor agonist is a confounder, as the effects on formation and/or function of β cells and α cells in stem cell–generated implants in patients is, at present, unknown. Aside from modest weight-lowering effects, GLP-1 receptor agonists appear to have limited direct effects on glycemia in patients with T1D^27^. After 9 months of liraglutide administration in this patient, body weight was 15% lower than at study entry while insulin dose was similar, as were the glucose control markers. However, at earlier timepoints (month 6 and month 9), insulin dose was lower than at baseline and was associated with improved glucose control measurements, which coincided with the established implant function. Our finding that the metabolic benefit at month 6 and month 9 was no longer observed at month 12 raises the question of whether liraglutide treatment interfered with the sustained glucose-controlling effect by the implant.

Of the six patients who did not achieve the primary efficacy endpoint (a change in baseline C-peptide response to MMTT at month 6), participation was discontinued for five before month 12 (cases 5, 7, 8, 9 and 10). Their CGM data at the latest available timepoint were compared with pre-implant values (Supplementary Table 3) and interpreted versus insulin dosing. For five of the six cases, the course in percent TIR reflected the insulin dosage: both remained unchanged (within 5% range) for cases 6, 9 and 10; both increased for case 5 or decreased for case 7. This contrasts with cases 1–3, which showed an increased TIR over time while insulin dosing decreased. Case 8 also exhibited an increased TIR after a higher insulin dose up to month 6; it was maintained at month 9 with a 10% lower insulin dose, which followed the 40% decrease in carbohydrate intake, known to reduce insulin needs. This patient’s general condition decreased during the study, showing signs of nephrotoxicity (up to threefold increase in creatinine levels) and bone marrow toxicity (severe anemia), which are known side effects of tacrolimus and mycophenolate mofetil. This illness led to a sedentary lifestyle and profoundly changed dietary habits.

A comparison of CGM measures averaged for the group that met efficacy endpoints for implant function (cases 1–3) with those averaged for the group that did not (cases 4–10) is less adequate to assess the glucose control improvement than a longitudinal analysis of individual cases, because it is limited by the low number of patients and by the inability to account for individual differences that affect outcomes. The median values of the selected CGM measures are, nevertheless, in line with the conclusions from the individual analyses (Supplementary Table 4). The group of cases 1–3 showed improvement over baseline in median TIR, TAR and GMI values at month 6 and month 9; these changes are, in aggregate, greater than those measured in the group of cases 4–10.

### Composition of stem cell–generated implants

Four retrieved sentinel devices were available for analysis of the composition of their inner chamber and of the tissue immediately outside the membranes. Two were retrieved from case 1 at month 6, and two were retrieved from case 4—one at month 3 and one at month 9.

**Fig. 3:**
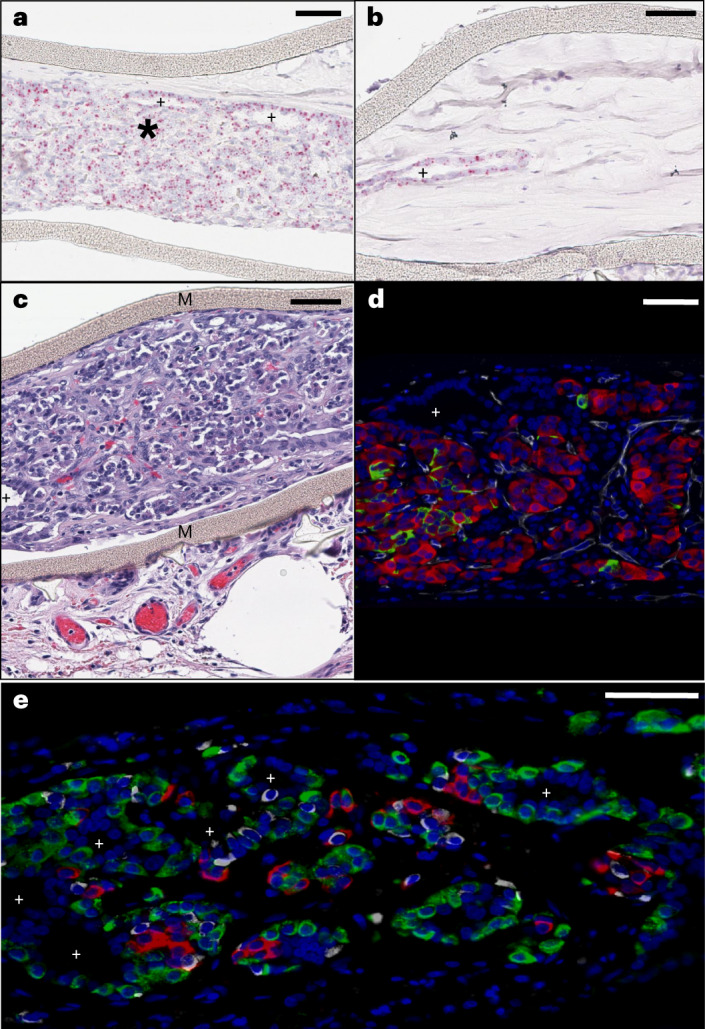
Composition of device-encapsulated human stem cell–derived implant at post-implant month 6. **a**–**e**, Histology of implant analyzed in sentinels retrieved from case 1 at month 6 and representative for the quantifications performed in the entire devices. **a**,**b**, RNA scope analysis for KDM5D positivity (red nuclei) identifying male donor cells in compact cell clusters and in cells lining cyst-like structures. The compact cell clusters (*, **a**) mainly contain endocrine cells. The cyst-like structures (+) are bordered by non-endocrine CK-positive cells as previously described^12,40^; they also occur in segments that are predominantly filled by KDM5D-negative fibroblasts embedded in connective tissue (**b**). **c**, Hematoxylin and eosin staining showing compact endocrine tissue with blood vessels (red erythrocytes) in the compartment defined by the membranes (M); a cyst-like structure surrounded by epithelial cells is also present (+). Larger blood vessels in loose connective tissue occur in the space outside the device membrane. **d**, Immunohistochemical stain for insulin (red), glucagon (green), CD34 (white) and DAPI (blue). **e**, Immunohistochemical stain for insulin (red), glucagon (green), somatostatin (white) and DAPI (blue). Small cyst-like structures can be observed in **d** and **e**. Scale bars, 50 µm.

Case 1 was a female recipient, which allowed identification of male donor cells by their KDM5D-positive nuclei. Both inner chambers contained segments that were mainly composed of donor cells and others that predominantly contained connective tissue embedding KDM5D-negative fibroblasts of recipient origin (Fig. 3). Both types of segments contained cyst-like structures, bordered by an epithelium of donor origin. The cell populations of donor origin represented 32% of the cells in the inner chambers, the result of low survival of the initially implanted cell mass (35% of cell mass at start) and of infiltration by recipient cells (representing twofold more cells) (Table 2).

**Table 2:** Cell composition of inner chambers in devices retrieved from T1D recipients during 12-month follow-up

	Case 1			Case 4			
	PT month 6			PT month 3		PT month 9	
	1	2	Average, % total		% Total		% Total
**Nuclear mass**							
DAPI-positive mass	3.3 µl	2.2 µl		4.6 µl		5.9 µl	
**Cell mass**							
Actin-positive	5.7 µl	3.8 µl		7.9 µl		10.0 µl	
% of cell mass at start	134%	89%	111%		186%		237%
CHRA-positive	1.5 µl	0.8 µl	24%	2.6 µl	33%	1.3 µl	13%
CK-positive	0.4 µl	0.3 µl	8%	1.0 µl	13%	1.1 µl	11%
ChrA-positive + CK-positive, % of cell mass at start			35%		86%		58%
Insulin-positive	0.3 µl	0.1 µl	3%	0.1 µl	1%	0.09 µl	<1%
% of cell mass at start			4%				
Glucagon-positive	0.8 µl	0.7 µl	16%	1.8 µl	22%	0.7 µl	7%

Volume measurements were conducted as outlined in Methods. PT, post-transplant.

Source data

Areas with packed donor cells exhibited insulin-positive cell clusters with associated glucagon-positive cells and adjacent CD34-positive cells forming capillaries (Fig. 3). Other areas mostly presented glucagon-positive cells and cytokeratin (CK)-positive cells that formed the epithelia of cyst-like structures. The insulin-positive cell mass represented 3% of the cell mass inside the devices and corresponded to 4% of the initial cell mass (Table 2). It was fivefold smaller than the glucagon-positive cell mass; their combined volume was smaller than the cell mass identified with the pan-endocrine marker Chromogranin A (CHRA), which can be attributed to the presence of poorly granulated β cells and other endocrine cell types (for example, somatostatin-positive cells).

Fibrous tissue of varying density and thickness was observed outside the device membranes, with more prominent blood vessels in looser layers (Fig. 3). Giant cell (CD68-positive) accumulations occurred around the structural mesh of the devices, sometimes with associated small lymphocyte clusters that were mainly composed of CD20-positive cells and less frequently with CD8-positive cells; immune cells were virtually absent inside the chambers (Supplementary Fig. 1). Donor cells were occasionally observed outside the chamber; their location was restricted to the space between the membrane and the structural mesh. In none of the sections was teratoma formation detected inside or outside the device chamber.

Analysis of the inner chambers from case 4, which achieved a C-peptide increase but not to 0.1 nmol l^−1^, indicated that recipient cells were already predominant over donor cells at month 3 and became more so at month 9 (Table 2). Donor cells had differentiated to insulin-positive and glucagon-positive cells as well as to hormone-negative, CK-positive cells lining duct-like structures. The mass of α cells formed was several-fold larger than that of the β cells. At month 9, the β cell mass represented less than 1% of the cells in the chamber, which is lower than in case 1, whereas total cell volume was higher.

Follow-up was discontinued before or at month 12 for six patients who did not achieve the primary efficacy endpoint of implant function. All devices were removed. The histologic analysis of tissue inside and outside the devices will be completed after their retrieval from all patients at the end of this cohort study.

## Discussion

The results presented here demonstrate that device-delivered stem cell–derived cells implanted in patients with T1D can establish a β cell mass that achieves sufficient function to improve glucose control. They support further development of hPSC-derived PECs as a β cell replacement therapy and the use of retrievable devices. This study was conducted with ViaCyte’s PEC-Direct combination product candidate consisting of PEC-01 cells in a device with perforated membranes administered in patients receiving immune suppression. Previous clinical work showed that functional β cells were formed in subcutaneous PEC-Direct implants but that their secretory capacity was too low for metabolic benefit^17–20^. The present study examined whether glucose control can be achieved by (1) increasing the cell dose through an increase in the number of implanted devices per patient and (2) using devices with the same pore configuration (placement and number) as that used in patients who demonstrated better engraftment in previous work^17,18^. We found that, in three of 10 patients, the function of the differentiated β cells was sufficient to improve glucose control markers from post-implant month 6 onwards. We attribute this outcome to formation of a larger β cell mass after delivery of a higher initial cell dose; it may also be related to higher cell survival due to the different pore configuration in the membranes^21^. Analysis of retrieved sentinel implants provided data on the size of the formed β cell mass and that of other cell types, which allows an assessment of the efficacy of the initial cell dose to replace a β cell mass as well as an identification of interfering processes.

The present study group had pre-transplant stimulated C-peptide levels below the LOD of 0.03 nmol l^−1^. The PEC-Direct implants increased this baseline level in four of 10 recipients at month 6, meeting the primary efficacy endpoint, and three of 10 (cases 1, 2 and 3) achieved levels >0.07 nmol l^−1^ that were sustained until month 12, a stable secondary efficacy endpoint for implant function. The levels in these three cases (≥0.10 nmol l^−1^) are considered metabolically relevant^19^. In cases 2 and 3, they are characteristic of a low functional state of β cells (0.1–0.2 nmol l^−1^), and, in case 1, they are characteristic of a state of intermediate function (>0.2 nmol l^−1^)^28^. The clinical relevance of inducing these C-peptide levels in patients with undetectable β cell function is also supported by their association with a beneficial complication profile^29^. This longitudinal and cross-sectional analysis of patients with T1D with different levels of residual insulin secretion found that C-peptide levels are inversely correlated with hypertension, HbA1c and cholesterol and also with microvascular complications^29^, extending previous observations^30,31^. This correlation has not yet been confirmed in immunosuppressed patients with an islet cell implant. Of direct clinical relevance is the observation that the three patients exhibited improved glucose control from month 6 onwards, as indicated by recently recommended measures of medical care in diabetes^23–25^. CGM data indicated marked improvement toward core endpoints for glucose control, approaching or achieving consensus targets (TIR > 70% and TAR < 25%), while lower exogenous insulin doses were used. The best control (TIR 85% at month 12) was established in the case with highest C-peptide response (case 1). Glycemic control and insulin production were also recently reported in two patients who received an intrahepatic stem cell–derived islet preparation (VX-880), one of whom became insulin independent with fasting or MMTT-stimulated C-peptide levels ≥0.16 nmol l^−1^ (ref. ^32^, poster presentation at the Annual Meeting of the American Diabetes Association (2023)).

Because inclusion in a study trial may itself influence outcomes both positively and negatively, we conducted a longitudinal analysis of individual patients. The association of a sustained C-peptide response ≥0.10 nmol l^−1^ together with sustained improvement in glucose control during reduced exogenous insulin dosing (cases 1–3), in the absence of confounding factors, indicates that the β cell mass in the implant exerted a glucose-controlling effect. Recipients who did not exhibit this level of β cell function (cases 4–10) did not show improvement in glucose control together with lower insulin dose. In case 5, better glucose control was observed after trial entry but was attributed to a higher insulin dosing. In case 8, side effects of the immunosuppressive agents altered lifestyle and led to a marked reduction in carbohydrate intake, thereby lowering glycemia independent of the implant. Overall, the data indicate that, when C-peptide levels ≥0.10 nmol l^−1^ were reached and maintained, a glucose-controlling effect was evident and that inclusion into the trial alone was not sufficient to achieve better glucose control at lower insulin dosage.

The best-performing PEC implant (case 1) did not achieve the criteria of good function as defined for intrahepatic implants of islet isolates from donor pancreases, such as a more than 50% reduction in insulin requirements^19^. However, its performance was markedly better than that reported for most extrahepatic islet cell implants^33–37^. In a comparative clinical cohort study on outcomes of extrahepatic or intrahepatic islet transplantation, extrahepatic implants (subcutaneous, gastric submucosal and omental) were independently associated with early graft failure; within the first 3 months, their stimulated C-peptide levels were significantly lower than those in intraportal implants (median 0.05 nmol l^−1^ versus 1.2 nmol l^−1^) and never exceeded 0.2 nmol l^−1^ (ref. ^37^). Among tested extrahepatic sites, the subcutis was associated with complete failure of human islet implants^37^ despite promising data in mice^38^. This again illustrates that successful outcome in animal models is not necessarily predictive for that in humans and supports the need for assessment in clinical investigational studies. The glucose-controlling effect that we have shown here is less pronounced than that in rodents, where a complete β cell replacement was established^39,40^. Further optimization is needed to increase efficacy of device-delivered hPSC-PECs to that of intrahepatic human islet implants. This can be guided by analysis of devices that are easily retrievable from the subcutaneous site. Implants of devices in an accessible site also helps meet safety concerns associated with stem cell–derived products. Our data will inform clinical studies on the safety and efficacy of cells that are genetically modified to promote their immune evasiveness and survival in allogeneic recipients (for example, ClinicalTrials.gov identifier: NCT05210530).

The present study provides in situ quantitative and qualitative analysis of a functional β cell implant in a cohort of patients with T1D. The data allow examination of correlations of initial cell dose with in vivo outcome as well as with similar measurements in preclinical models. Extrapolation of analysis in exploratory sentinel devices to the large devices is supported by the similarity in composition of small and large device chambers^17^. The data can be compared with those obtained from similar sentinel human embryonic stem cell (ESC)-derived-PE implants in immunocompromised nude rats. In case 1, with the best in vivo outcome in this study, the size of the β cell mass (0.2 µl) is 12-fold to 18-fold smaller than that in devices retrieved from rats in which the hu-ESC-PE implant achieved glucose-induced plasma hu-C peptide >1 nmol l^−1^ (Supplementary Table 5). This difference can only be partly attributed to an earlier time of analysis (post-transplant month 6 versus month 14 in rats). Quantification of pancreatic cell types in the retrieved patient sentinels indicated that their cell mass represented only 35% of that at the start versus more than 200% in sentinels from rats (Supplementary Table 5). This lower recovery can be attributed to a higher cell loss in the device, possibly the result of a more invasive surgical procedure, a stronger foreign body reactivity in humans and/or a slower wound healing and capillary ingrowth in a patient with T1D. In addition to a higher loss of donor cells in the clinical setting, the degree of β cell differentiation of surviving cells may also have been lower than in sentinels of rat recipients. When extrapolating the β cell mass measured in sentinels of case 1 to the entire PEC-Direct implant in this patient, it amounted to ~0.24 µl kg^−1^ body weight, which is 25-fold lower than that established in subcutaneous open devices placed in nude rats (Supplementary Table 5).

The insulin-positive cells occur clustered with adjacent capillaries, a characteristic of functional secretory units responsible for glucose-responsive release of C-peptide and proinsulin. The sustained elevation of proinsulin levels during prolonged glucose stimulation indicates a continued activation of β cells to provide newly synthesized hormone during this metabolic demand, whereas the progressive decline in C-peptide expresses a shortage of the hormone reserves^22^. These data are characteristic for a β cell mass of insufficient size, which has to recruit all cells into sustained synthetic and secretory activity instead of maintaining subpopulations that build stores of fully processed hormone for use during sustained demands^41^. Depletion of this store explains the insufficient correction of the MMTT-induced hyperglycemic state. An increased plasma proinsulin to C-peptide ratio was also observed in patients receiving an intrahepatic islet transplant and was related to an insufficient β cell mass^42^, also related to MMTT-induced hyperglycemia^43^. The circulating hormonal markers plasma proinsulin and C-peptide can be used to evaluate clinical studies that aim to achieve a larger β cell mass.

The devices that we used offer sufficient space for formation of a large β cell mass as indicated by our data in nude rats. Improvements are needed to promote better cell survival and differentiation to β cells. Our analysis indicated that α cell formation was favored. Glucagon-positive cell mass was fivefold higher than the insulin-positive cell mass, a twofold higher ratio than that seen in rodent implants with metabolically adequate β cell function^12,40^. These glucagon-positive cells are expected to support growth and function of neighboring β cells^44^, but they can also increase plasma glucagon levels as detected in rodent models^12,39,40^, the impact of which is so far unknown. It will also be necessary to limit ingrowth of recipient fibroblasts and associated connective tissue formation, as the space they occupy within the devices becomes unavailable to the graft and may interfere with cell differentiation. The issue of fibroblast infiltration is evident from the data collected in case 4, which showed marginal C-peptide levels, a very low proportion of hormone-positive cells and a high proportion of infiltrating recipient cells. The possible causes for outcome differences in the 10 patients, including differences in the degree of donor cell survival and differentiation to β cells and in the degree of ingrowth of fibroblasts or endothelial cells, warrant further investigation. This analysis of implants in the context of circulating glucose, C-peptide and proinsulin levels is informative, particularly for the case with the best functional outcome. The data of this pilot study support inclusion of these assessments in future protocols.

We attribute the achievement and maintenance of higher C-peptide responses than in the previous PEC-Direct studies^17,18^ to the formation of a larger β cell mass after implantation of a twofold to threefold higher initial cell dose. The device membranes also had a different pattern of perforations, which may have influenced capillary ingrowth in the implanted cell mass and, hence, formation and differentiation of β cells. Vascularization may still be insufficient in timing and density to form and support a metabolically adequate functional β cell mass. Further device optimization seems preferred for improving outcomes than further increasing the number of devices and initial cell dose. In the present study, the implanted cell dose was, on average, 14 × 10^6^ cells per kilogram of body weight, which is in the range of doses reported for intrahepatic islet cell implants (6–18 × 10^6^ cells per kilogram of body weight; refs. ^1,2^). It resulted in a β cell mass that occupied less than 5% of the inner chamber of the devices that we examined. Addition of an angiogenic component may be considered in view of observed effects on engraftment and function of β cells in extrahepatic sites of rodent recipients^45–47^. Such an approach may help donor cell survival and endocrine differentiation and reduce the size of fibrotic areas.

In conclusion, this report shows the feasibility of achieving glucose control by a stem cell–generated β cell mass in a retrievable device placed subcutaneously in patients with T1D. Data from retrieved devices relate in vivo outcome to the formed β cell mass and indicate processes for improving efficacy of the device.

## Methods

### Global description of clinical trial

This trial is a first-in-human, phase 1/2, open-label study on safety, tolerability and efficacy of VC-02 in patients with T1D and hypoglycemia unawareness. VC-02 is a combination product of PEC-01 cells loaded into a delivery device. The study is conducted at nine centers in North America and one center in Belgium (ClinicalTrials.gov identifier: NCT03163511). This paper is an interim report on one cadre of 10 patients in the larger study. The patients were treated at City of Hope; the University of British Columbia; the University of California, Davis; the University of Minnesota; and Vrije Universiteit Brussel. A detailed description of the clinical protocol is provided as a Supplementary Protocol.

Inclusion criteria included men and non-pregnant women, a diagnosis of T1D for a minimum of 5 years, hypoglycemia unawareness (Clarke score) or significant glycemic lability, a stable diabetes treatment regimen, willingness to use a CGM device and being an acceptable candidate for surgical implantation. A Clarke score ≥4 confirmed patients qualifying for study participation on the basis of hypoglycemia unawareness. Exclusion criteria included history of islet cell, kidney and/or pancreas transplant; six or more severe unexplained hypoglycemic events within 6 months of enrollment; uncontrolled or untreated thyroid disease or adrenal insufficiency; diabetic complications, such as severe kidney disease or renal dysfunction, proliferative retinopathy, foot ulcers, amputations and/or severe peripheral neuropathy; or detectable stimulated serum C-peptide during the screening period defined as ≥0.07 nmol l^−1^.

After establishing a safety profile of the product candidate in cohort 1, product efficacy was evaluated in cohort 2. Within cohort 2, patients were enrolled in cadres (groups of patients ranging from *n* = 4 to *n* = 11) for testing a particular device configuration and/or implant strategy to improve product engraftment and cell survival outcomes. All device configurations contained the same materials but differed in the application of the pores. Differences in implant strategy involved pharmacological interventions, number of dose-finding units implanted and/or implant sites. The patients described in refs. ^17,18^ had been enrolled in earlier study groups of cohort 2. Those described in the present report participated in the most recent one (enrollment between August 2020 and October 2021); they received devices with the same materials as the devices in previous study groups but in twofold to threefold higher number and with a different application of perforations (described in patent application US16/347,790). The protocol specified the primary efficacy endpoint as change from baseline to week 26 in plasma C-peptide after MMTT and the following secondary efficacy endpoints for follow-up to maximally 104 weeks:

- Change from baseline of C-peptide response to MMTT and percent of patients achieving levels >0.07 nmol l^−1^;
- Change from baseline in average insulin dose and percent of patients with 50% reduction and percent with insulin independence;
- Percent of time with blood glucose values <54 mg dl^−1^, 54 mg dl^−1^ to <70 mg dl^−1^, 70 mg dl^−1^ to ≤180 mg dl^−1^ and >180 mg dl^−1^ (CGM device) and change from baseline in time-in-hypoglycemic range (<70 mg dl^−1^), time-in-euglycemic range (70–180 mg/dl^−1^) and time-in-hyperglycemic range (>180 mg dl^−1^);
- Frequency of hypoglycemic events (HEs) and percent of patients free of HE.

### Clinical protocol

Patients fulfilled entry criteria including hypoglycemia unawareness, plasma C-peptide <0.07 nmol l^−1^ during MMTT, HbA1c ≤10% and signed informed consent. The clinical protocol used immune suppression with anti-thymocyte globulin (ATG) for induction, and tacrolimus and mycophenolate mofetil for maintenance as in refs. ^17,18^. ATG dose was adjusted to lymphocyte counts. Two days after initiation of ATG, devices were placed subcutaneously in the abdominal wall and flanks, under general anesthesia. No adverse events were recorded during and after the interventions.

As required by the study protocol, all CGM data were acquired by a Dexcom G6 CGM sensor. Glycemic control and insulin administration were guided by a diabetes educator who was independent of the study team and unaware of plasma C-peptide levels. Regular hospital visits were scheduled for medical follow-up, including systematic survey for adverse events, collection of laboratory data and adjustment of immune suppression dosing when needed. The CGM Medtronic automodus was stopped at least 4 h before start of MMTT, and the patient was switched to a fixed insulin dose that was maintained during the test. The mixed meal consisted of Boost Hi-Protein (volume, 360 ml) and was ingested within 15 min. No insulin bolus was given before the meal.

An MMTT was carried out 4 weeks before transplant and at 3-month post-transplant intervals. Blood was collected every 30 min for measurement of glycemia and C-peptide, with the purpose of identifying recipients who increased plasma C-peptide between baseline and month 6 (primary efficacy endpoint) and those who achieved a level >0.07 nmol l^−1^ (secondary efficacy endpoint) during a 12-month follow-up. The 90-min timepoint in the MMTT was selected for this assessment, as conventionally done; concurrent glycemia was >250 mg dl^−1^ for all patients and, thus, considered as a similar glucose-stimulatory condition for all. Glucose responsiveness was evaluated by comparing 0-min and 90-min C-peptide values. For the endpoint of case 3 (month 12), this glycemia criterion was reached only at minute 120, thus providing the listed data. Follow-up was extended to 360 min for the month 9 and month 12 MMTT of the case with highest C-peptide response at month 6 to follow release of both C-peptide and proinsulin during prolonged glucose stimulation.

Glucose, C-peptide and HbA1c levels were analyzed by ACM Global Laboratories using hexokinase, chemiluminescent and ion exchange high-performance liquid chromatography assays, respectively. For one point (case 3, month 9), the CRO value for C-peptide was undetectable, differing with the local hospital value (0.10 nmol l^−1^) as well as with the prior and following CRO values in this patient, such that we think that it is erroneous and can be replaced by the locally measured value. Proinsulin was measured only in the MMTT samples of case 1, month 12; values were obtained by digital ELISA technology (SIMOA HD1, Quanterix). As in previous protocols, start of immune suppression caused a transient decrease in blood cell counts and HbA1c level.

### Implantation of PEC-Direct

The PEC-Direct combination product was manufactured by ViaCyte as perforated device units containing PEC-01 that had been differentiated from the CyT49 pluripotent stem cell line^6,16^. The cells were loaded in dose-finding and in sentinel units where they were retained between polytetrafluoroethylene membranes with pores in controlled distribution, number and diameter. The density of pores is identical for both devices, as is thickness; therefore, engraftment, diffusion and perfusion are expected to be similar. The scaling of the dose-finding device is 12× for area containing cells; they are estimated to contain 75 × 10^6^ PEC-01 cells versus 7 × 10^6^ PEC-01 cells in the sentinels. The sentinel devices were introduced in the same implant site, on the opposite end of an incision used to place the dose-finding device. These units were prepared at ViaCyte (ref. ^16^ and Supplementary Information) and shipped to the implant centers within a container that maintains 15–25 °C for up to 5 d.

### Histology

Retrieved sentinels with surrounding tissue were fixed in formaldehyde before embedding in paraffin and sectioning (5 µm). For histology, sections were stained with hematoxylin and eosin. To distinguish male donor cells from female recipient cells (case 1), the RNAscope 2.5 HD Assay RED (cat. no. 322350, Advanced Cell Diagnostics) was used according to the manufacturer’s instructions (15 min, Target Retrieval; 30 min, Protease Plus). The probe used was *Homo sapiens* KDM5D (cat. no. 558161), a gene located on the Y chromosome. Because the CyT49 cell line is of male origin and the recipient is female, KDM5D-positive (red) signals distinguish donor from recipient cells. RNA in situ hybridization was performed at the VSTA facility of Vrije Universiteit Brussel https://vsta.research.vub.be.

For immunohistochemical analysis, sections underwent heat-induced antigen retrieval (2100-Retriever, Aptum Biologics) in citrate buffer (ScyTek Laboratories) before staining with guinea pig anti-insulin and rabbit anti-glucagon (each 1:1,000, in-house produced), mouse anti-glucagon (1:500, g2654, Sigma-Aldrich), rat anti-somatostatin (1:100, ab30788, Abcam), mouse anti-CK19 (1:20, M0888, Agilent), rabbit anti-wide spectrum CK (1:200, z0622, Agilent), mouse anti-CHRA (1:500, Ma5-13287, Thermo Fisher Scientific) and rabbit anti-CD34 (1:150, ab81289, Abcam). Alexa Fluor-conjugated F(ab′)_2_ fragments of affinity-purified antibodies were used as secondary antibodies allowing multiple labeling (1:500, Jackson ImmunoResearch). Nuclei were stained by DAPI (Sigma-Aldrich) added to the mounting medium (Agilent). Digital images were acquired on an Axioplan 2 microscope (Carl Zeiss) with an Orca-R2 camera (Hamamatsu Photonics) and SmartCapture 3 software (DSUK Ltd.) or acquired with an Aperio CS2 (Leica Biosystems) and visualized with Pathomation software. Morphometry was conducted on .tiff pictures captured with ImageXpress Pico (Molecular Devices) and semi-automatically analyzed with IPlab software (Becton Dickinson).

The composition of the tissue in the inner chamber was determined as described in ref. ^40^. In previous work, we determined the number of evenly distributed sections that are representative for this type of implant. This number was stained for DAPI (marker for nuclear mass) and for insulin, glucagon and CHRA and CK (cytoplasmic pancreatic cell markers). For each staining, the positive area in a section was semi-automatically quantified and extrapolated to a total volume for the specified marker according to the Cavalieri method: the value was multiplied by the distance to the next section, which yields a volume for that region, and all region volumes were added to obtain the total volume for the implant. Nuclear mass was also expressed as percent of that at start (2.5 µl) as well as translated to total cellular mass by applying a conversion factor that was previously defined in stem cell preparations stained for DAPI and actin. This total cellular mass consists of the mass of infiltrated recipient cells and that of the pancreatic cell types of donor origin. Insulin-positive and glucagon-positive cells were CHRA positive. The sum of CHRA-positive mass and CK-positive mass was taken as mass of donor origin; its percentage over total cell mass allowed calculation of the recovery of initial cell mass as well as of the proportion of infiltrating recipient cells (Table 2).

The presence and phenotype of lymphocytes were identified by immunohistochemical staining for CD4 (SP35), CD8 (SP57), CD20 (L26) and CD68 (KP1). Slides were scanned with an Aperio GT450 at ×40 magnification with visualization with Pathomation software.

### Ethics statement

All patients signed an informed consent, which also stated the existence of other treatment forms to be explained by the study physician before taking a decision. The consent included an agreement on collection and use of study data for research purposes and in connection with scientific and medical publications. All study sites were approved by the local institutional review board associated with their institution. The trial was conducted in compliance with all applicable regulations.

### Reporting summary

Further information on research design is available in the Nature Portfolio Reporting Summary linked to this article.

## Online content

Any methods, additional references, Nature Portfolio reporting summaries, source data, extended data, supplementary information, acknowledgements, peer review information; details of author contributions and competing interests; and statements of data and code availability are available at 10.1038/s41587-023-02055-5.

## Supplementary information

Supplementary Information Supplementary Tables 1–5 and Supplementary Fig. 1.

Reporting Summary

Supplementary Data Clinical protocol

## Source data

Source Data Table 2 Source data for Table 2: Analysis of cell composition of inner chambers in devices retrieved from T1D recipients.

## Data Availability

Source data for Table 2 are provided. Morphometric data not included in this paper are stored at Vrije Universiteit Brussel and are available upon reasonable request to D.P. (daniel.pipeleers@vub.be). Source data are provided with this paper.
