# Supplementary material for: Encapsulated stem cell–derived β cells exert glucose control in patients with type 1 diabetes
Source: Nat Biotechnol. 2023 Nov 27;42(10):1507–14. doi: 10.1038/s41587-023-02055-5 (PMC11471599; doi:10.1038/s41587-023-02055-5)
Supplement: Supplementary file 1 — Supplementary Tables 1–5 and Supplementary Fig. 1. [file 41587_2023_2055_MOESM1_ESM.pdf]

nature biotechnology

Article

https://doi.org/10.1038/s41587-023-02055-5

Encapsulated stem cell–derived β cells exert glucose control in patients with type 1 diabetes

In the format provided by the authors and unedited

Supplementary Information

Supplementary Table 1. Patient characteristics at time of implantation and start immune suppression

Case	Gender	Age		Weight	BMI	Insulin pump PreTx	Insulin Dose*		Period PreTx	Hypogl.Events		GMI	HbA1c	Immune Suppression Treatment				
		onset T1D	Baseline	Baseline	Baseline		Baseline mean		Dexcom G6	Number preTx		Baseline	Baseline	Thymoglobulin®		Lymph.count	Tacro	MMF
		yrs	yrs	kg	kg/m^2		IU/day	IU/kg/day	days	Total	per week	%	%	Start	Total Dose	D1	Start	Start
															mg/kg	10^3/uL		1 g/day
1	F	15	51	68.6	23.2	Medtronic 670G	18.4	0.27	15	0	0.00	7.7	7.4	D -2	2.9	0.2	D -10	D -10
2	M	40	48	71	23.7	No	43.1	0.61	30	17	3.97	8.0	8.0	D -2	4.5	0.1	D -6	D -6
3	F	14	41	60.5	19.8	Medtronic 630G	27.1	0.36	30	13	3.03	7.3	8.5	D -2	3.0	0.2	D -1	D -1
4	M	40	57	85.8	30.8	No	65.2	0.76	32	2	0.44	7.3	6.8	D -2	2.9	0.2	D -1	D -1
5	M	13	61	87.3	27.2	No	57.1	0.65	18	8	3.11	7.3	7.9	D -2	2.9	0.4	D -5	D -5
6	M	27	63	59.2	21.2	No	28.3	0.48	31	23	5.19	6.5	6.1	D -2	2.9	0.2	D -1	D -1
7	F	5	59	47.5	20.5	Medtronic 630G	25.8	0.54	30	0	0.00	8.1	8.4	D -2	3.1	0.3	D -1	D -1
8	F	3	50	68.3	27.7	Omnipod	45.5	0.67	30	0	0.00	7.8	7.4	D -2	2.9	0.1	D -7	D -7
9	F	10	48	78.3	27.6	Omnipod	26.2	0.33	23	11	3.35	7.1	8.0	D -2	2.9	0.3	D -7	D -7
10	M	10	36	89.2	23.7	Tandem Control IQ	37.1	0.42	30	1	0.23	6.7	6.8	D -2	2.9	0.2	D -1	D -1

Patients are ranked as in Table 1. PreTx mode of insulin treatment was maintained PostTx * mean during minimally 2 weeks pre-implant
Hypoglycemic events from CGM and/or patient-reported GMI= Glucose Management Indicator

These patients entered, and were followed for this study at City of Hope, The University of British Columbia, University of California-Davis, University of Minnesota, Vrije Universiteit Brussel (alphabetical order)

Supplementary Table 2. Metabolic Parameters before (Pre) and following PEC-Direct Implant in recipients reaching the primary efficacy endpoint

Post-implant data of CGM, Insulin dose, Carbohydrate intake were collected over 3-months prior to the quarterly end points. Pre- data cover the last two to four weeks. Number of severe hypoglycemic events at screening cover 12 months Pre, 3-month periods Post-implant. Other data are determined at indicated time points. N.A.= not available N.D.= not determined

Case 3 received Liraglutide from 2 weeks before month 3, started at 0.6 mg/day, progressively increased to 3 mg/day at month 6, followed by 3 doses of 3.6 mg/week till month 12.

	Case 1					Case 2					Case 3					Case 4				
	Pre	**Mo 3**	**Mo 6**	**Mo 9**	**Mo 12**	***Pre***	**Mo 3**	**Mo 6**	**Mo 9**	**Mo 12**	***Pre***	**Mo 3**	**Mo 6**	**Mo 9**	**Mo 12**	***Pre***	**Mo 3**	**Mo 6**	**Mo 9**	**Mo 12**
**CGM Data**																				
Number of days recorded	*15*	81	98	91	91	*30*	90	90	90	90	*30*	90	90	90	90	*32*	81	77	112	91
% of time recorded	*95*	94	99	99	98	*96*	95	95,9	96,9	97,4	*82.5*	87.4	92.7	85.6	84.7	*93*	98	98	97	97
Mean glycemia (mg/dl)	*182*	171	157	158	144	*211*	187	167	152	155	*169.2*	156.6	144	145.8	158.4	*165*	148	147	161	158
Gluc. Manag. Indicator (GMI) (mmol/mol)	*60.2*	57.3	53.8	54.0	50.4	*67.9*	61.6	56.4	52.4	53.2	*56.9*	53.7	50.4	50.8	54.1	*55.9*	51.5	51.1	54.7	53.9
(%)	*7.7*	7.4	7.1	7.1	6.8	*8.4*	7.8	7.3	6.9	7	*7.4*	7.1	6.8	6.8	7.1	*7.3*	6.9	6.8	7.2	7.1
Glycemic variability (% CV)	*30*	29	32	28	27	*40.4*	40.9	38.6	37.7	37.7	*29.7*	31.6	31.4	30.3	31.3	*35*	39	37	38	39
Percent readings																				
Time above range (TAR): > 250 mg/dl	*11*	7	6	4	1	*33*	22	11	6	6	*7*	6	2	2	4	*10*	6	5	9	9
181-250 mg/dl	*33*	30	19	22	13	*28*	27	29	23	24	*30*	25	18	17	27	*27*	21	20	26	23
Time in range (TIR): 71-180 mg/dl	*55*	63	74	74	85	*36*	48	56	67	66	*62*	68	79	80	68	*59*	68	71	61	63
Time below range (TBR): 54-70 mg/dl	*0*	0	1	0	1	*3*	2	3	3	3	*1*	1	1	1	1	*3*	5	4	3	4
< 54 mg/dl	*0*	0	0	0	0	*0*	1	1	1	1	*0*	0	0	0	0	*1*	0	0	0	1
Number of severe hypoglycemic events	0	0	0	0	0	0	0	0	0	0	1	0	0	0	0	0	0	0	0	0
**Mean Insulin Dose**																				
Number of days recorded	*15*	81	90	90	90	*30*	90	90	90	90	*30*	90	90	90	90	*22*	80	77	111	92
Total insulin (mean IU/day)	*18.4*	29.5	34.7	30.6	19.9	*46.5*	46.5	42.5	42.7	40.2	*27.1*	28.9	21.4	16.6	22.8	*88.5*	88.3	80.5	77.5	77.1
Basal insulin (mean IU/day)	*9.2*	12.9	15.5	12.8	9.9	*22.9*	23.5	20	17.2	23.7	*20.4*	18.7	14.9	10.9	12.6	*46.5*	47.4	45.9	45.0	46.0
(% total)	*50*	44	45	42	50	*49*	51	47	40	59	*75*	65	70	66	55	*53*	54	57	58	60
Bolus insulin (mean IU/day)	*9.2*	16.6	19.2	17.8	10.0	*23.6*	22.9	22.5	25.5	16.5	*6.7*	10.2	6.5	5.7	10.2	*42.0*	40.9	34.6	32.5	31.1
(% total)	*50*	56	55	58	50	*51*	49	53	60	41	*25*	35	30	34	45	*47*	46	43	42	40
Total insulin (mean IU/day/kg)	*0.27*	0.40	0.46	0.40	0.26	*0.65*	0.63	0.59	0.57	0.55	*0.45*	0.45	0.37	0.32	0.44	*1.03*	1.04	0.98	0.93	0.94
**Daily carbohydrates** (g mean ± SD)	*86 ± 50*	185 ± 70	185 ± 81	185±82	113±85	N.A.	N.A.	N.A.	N.A.	N.A.	N.A.	N.A.	N.A.	N.A.	N.A.	*N.A.*	N.A.	N.A.	N.A.	N.A.
**Body weight** (kg)	*68.6*	73.0	75.2	76.6	77.0	*71.5*	74	72	74.4	72.8	*60.5*	64.5	57.3	51.3	51.3	*85.8*	84.6	82.4	83.2	81.8
**BMI** (kg/m$^{2)}$	*23.2*	24.7	25.4	25.9	26.0	*24.6*	25.5	24.8	25.6	25.1	*19.8*	22.2	18.7	17.7	17.7	*30.8*	30.3	29.6	29.8	29.3
**Serum creatinine** (μmol/L)	*72*	74	71	69	75	*76*	73	96	78	89	*84*	80	86	81	87	*91*	114	105	113	116
**Hematocrit** (L/L)	*0.34*	0.35	0.35	0.37	0.37	*0.47*	0.37	0.34	0.40	0.37	*0.38*	0.41	0.41	0.38	0.38	*0.39*	0.35	0.37	0.37	0.38
**Hemoglobin** total (g/L)	*108*	109	114	112	112	*159*	124	117	133	126	*124*	133	138	127	124	*137*	118	121	117	125
**HbA1c** (%)	*7.4*	6.4	6.9	6.6	6.9	*8*	6.7	6.1	N.D.	6.1	*8.5*	N.D.	6.7	N.D.	6.9	*6.8*	5.8	5.5	6.0	5.9

Supplementary Table 3. Metabolic Parameters before (Pre) and following PEC-Direct Implant in recipients not reaching the primary efficacy endpoint

Post-implant data of CGM, Insulin dose, Carbohydrate intake were collected over 3-months prior to the quarterly end points. Pre- data cover the last two to four weeks. Other data are determined at indicated time points. N.A.= not available N.D.= not determined

CGM percent readings TAR > 180 mg/dl, TIR 71-180 mg/dl, TBR > 70 mg/dl

	**Case 5**				**Case 6**					**Case 7**			
	Pre	**Mo 3**	**Mo 6**	**Mo 9**	***Pre***	**Mo 3**	**Mo 6**	**Mo 9**	**Mo 12**	***Pre***	**Mo 3**	**Mo 6**	**Mo 9**
**CGM Data**													
Mean glycemia (mg/dl)	*173*	151	154	157	*135*	161	136	136	137	*205*	182	182	209
Gluc. Manag. Indic. (GMI) (%)	*7.4*	6.9	7.0	7.1	*6.5*	7.2	6.6	6.6	6.6	*8.2*	7.6	7.7	8.3
Glycemic variability (% CV)	*31.2*	36.6	36.5	33.5	*40*	37	35	32	34	*40.5*	37.5	38.5	34.5
Percent readings													
Time above range (TAR)	*42*	25	27	30	*20*	33	17	15	18	*55*	46	48	62
Time in range (TIR)	*57*	73	71	68	*73*	64	80	83	78	*43*	52	50	37
Time below range (TBR)	*1*	2	2	2	*7*	2	4	2	4	*2*	2	2	1
**Insulin dose** (IU/day/kg)	*0.71*	0.80	0.82	0.81	*0.48*	0.51	0.52	0.54	0.51	*0.60*	0.50	0.42	0.44
**Daily carbohydrates** (g mean)	*N.A.*	N.A.	N.A.	N.A.	*N.A.*	N.A.	N.A.	N.A.	N.A.	*N.A.*	N.A.	N.A.	N.A.
**Body weight** (kg)	*87.3*	86.2	85.9	84.0	*59.2*	59.0	59.6	59.4	59.6	*47.5*	49.5	52.2	52.1
**Serum creatinine** (µmol/L)	*85*	111	105	106	*68*	110	105	98	97	*74*	80	99	87
**Hemoglobin** total (g/L)	*128*	124	119	125	*134*	120	126	129	127	*127*	107	102	107
**HbA1c** (%)	*7.8*	N.D.	6.8	N.D.	*6.1*	N.D.	5.5	N.D.	5.6	*8.4*	N.D.	7.1	N.D.

	**Case 8**				**Case 9**				**Case 10**		
	Pre	**Mo 3**	**Mo 6**	**Mo 9**	***Pre***	**Mo 3**	**Mo 6**	**Mo 9**	***Pre***	**Mo 3**	**Mo 6**
**CGM Data**											
Mean glycemia (mg/dl)	*171*	167	146	104	*173*	166	176	176	*145*	135	141
Gluc. Manag. Indic. (GMI) (%)	*7.4*	7.3	6.8	6.7	*7.5*	7.3	7.5	7.5	*6.8*	6.5	6.7
Glycemic variability (% CV)	*33.8*	32.2	36.4	34.5	*45.6*	40.9	41.1	43.9	*27.3*	31.7	32.3
Percent readings											
Time above range (TAR)	*37*	36	21	17	*36*	34	43	40	*15*	14	18
Time in range (TIR)	*63*	63	77	81	*60*	63	54	57	*83*	83	80
Time below range (TBR)	*0*	1	2	2	*4*	3	3	3	*2*	3	2
**Insulin dose** (IU/day/kg)	*0.52*	0.61	0.58	0.53	*0.39*	0.40	0.40	0.35	*0.45*	0.47	0.45
**Daily carbohydrates** (g mean)	*162*	192	128	94	*N.A.*	N.A.	N.A.	N.A.	*N.A.*	N.A.	N.A.
**Body weight** (kg)	*68.3*	72.2	75.0	74.0	*78.3*	77.4	77.2	78.5	*89.2*	88.0	89.5
**Serum creatinine** (µmol/L)	*60*	84	117	172	*73*	89	79	65	*66*	81	76
**Hemoglobin** total (g/L)	*123*	86	108	99	*127*	116	115	114	*146*	138	149
**HbA1c** (%)	*7.4*	N.D.	6.2	N.D.	*8.0*	N.D.	7.5	N.D.	*6.8*	N.D.	6.4

Supplementary Table 4. Comparison of changes in CGM-endpoints for glucose control measured in the group of cases that meet efficacy endpoints for implant function with those in the group of cases that do not meet these endpoints

**Efficacy Endpoints**	**TIR (%)**			**TAR (%)**			**GMI (%)**		
**Implant Function***	**Pre-Tx**	**month 6**	**month 9**	**Pre-TX**	**month 6**	**month 9**	**Pre-Tx**	**month 6**	**month 9**
**Yes : Cases 1-3**	**55** (36; 62)	**+ 19** (+17; +20)	**+ 19** (+18; +31)	**44** (37; 61)	**- 19** (-21; -17)	**- 18** (-32; -18)	**7.7** (7.4; 8.4)	**- 0.6** (-0.9; -0.6)	**- 0.6** (-1.5; -0.6)
**No : Cases 4-10**	**60** (57; 73)	**+ 7** (-3; +14)	**+ 6** (-4; +13)	**37** (20; 42)	**- 7** (-15; +3)	**- 4** (-14; +5)	**7.4** (6.8; 7.5)	**- 0.4** (-0.5; 0)	**- 0.1** (-0.4; +0.1)
		P = 0.008	P = 0.036		P = 0.017	P = 0.095		P = 0.042	P = 0.083

* MMTT-induced plasma C-peptide > baseline at month 6 with levels ≥ 0.1 nmol/l up to month 12.
median (Q1;Q3) values for baseline (Pre-Tx) and for changes versus baseline (n=10 at month 6, n=9 at month 9)
comparison of groups by two-tailed, unpaired Mann-Whitney tests; statistical significance for P ≤ 0.05

Supplementary Table 5.

Outcome of device-delivered stem cell-generated beta cell implants was examined in immune-compromised nude rats following the same experimental design as for device-encapsulated hu-PSC-PE implants in immune-compromised mice (12, 39). Protocol and procedures were approved by the Ethical Committee of VUB and carried out according to European Community Council Directive (86/609/EEC). Each rat received a subcutaneous open device that had been filled with 7.10^6 hu-ESC-PE cells at ViaCyte before shipment to Brussels where the study was conducted. Recipients were followed for appearance of plasma human (hu)-C-peptide and glucose-dependent changes in this level. A group of five rats were followed over 55 to 60 weeks before retrieval of the device for histological analysis as described in Methods and in previous reports (39, 40). The composition of the inner chamber was examined as for devices from cases 1 and 4, with measurement of the cell mass that corresponds to donor cells and of the insulin-positive cell mass. Rats are ranked according to their plasma hu-C-peptide levels at min 60 following an intraperitoneal (IP) glucose load between PT-week 40 and 60; three IP-glucose tests were performed during this period. The percent recovery of donor cells expresses the balance between cell loss following implantation and expansion of surviving cells. Expansion of donor cells in implants 3 to 5 contributed to a 2- to 4-fold higher donor cell mass than at start, with formation of an insulin-positive cell mass that was 12- to 18-fold higher than that in the month 6-device of case 1. Data confirm variability in donor cell survival and beta cell formation in subcutaneous devices containing hu-PSC-PE cells (39, 40).

Nude Rat	IP-glucose-induced plasma hu-C-peptide PT wk 40-60	Donor cell mass at PT wk 55-60		
		**Relative to cell mass at start**	**Insulin-positive cell mass**	
	min 60 nmol/l	%	µl	µl / kg BW
**1**	< LOD = 0.05	0	0	0
**2**	0.5, 0.7, 0.5	103	0.85	1.44
**3**	0.7, 1.3, 1.6	232	3.02	6.25
**4**	3.8, 1.7, 2.6	271	3.64	6.74
**5**	2.4, 6.7, 2.4	380	2.51	4.56

Supplementary Figure 1. Lymphocyte phenotypes in implants at Month 6 from case 1

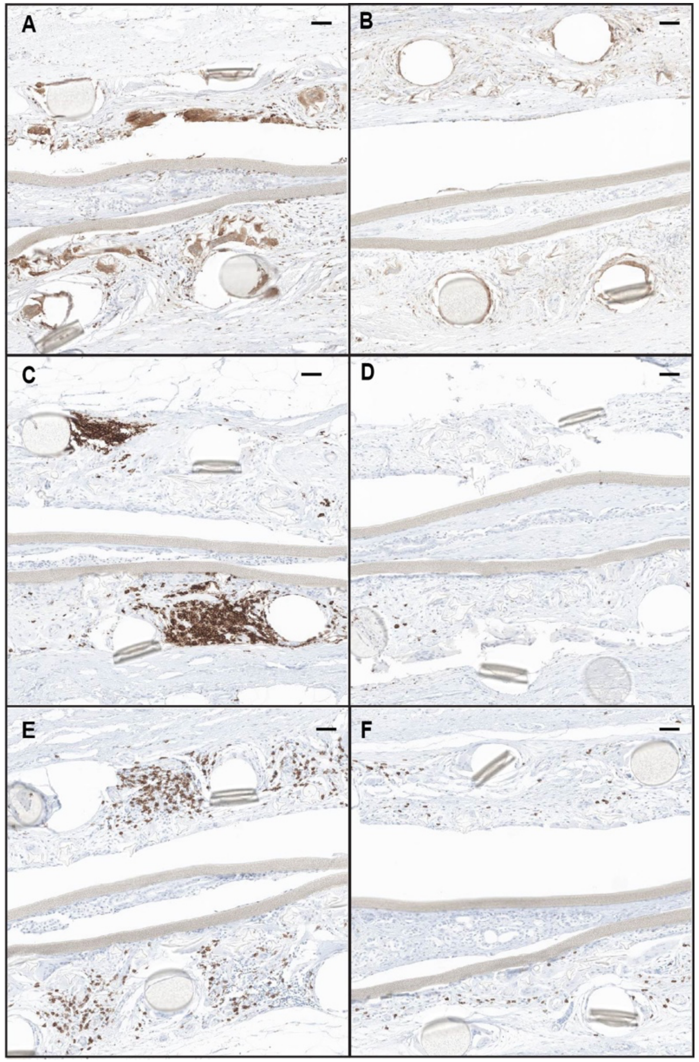

Immune-staining for CD4, CD8, CD20, CD68-positive cells indicated that the inner chamber exhibited a low density of single lymphocytes without signs of accumulation (A-F). The tissue outside the chamber presented CD68-positive cells clustered around the structural mesh, with varying density (A, B); in few segments, these clusters were associated with CD20-positive clusters (C,D), and less frequently with CD8-positive cells (E,F). CD4-positive cells were rarely observed and never occurred in clusters. These observations were made in each of the two retrieved devices.

Scale bar = 50 μm.
