# Supplementary material for: Encapsulated stem cell–derived β cells exert glucose control in patients with type 1 diabetes
Source: Nat Biotechnol. 2023 Nov 27;42(10):1507–14. doi: 10.1038/s41587-023-02055-5 (PMC11471599; doi:10.1038/s41587-023-02055-5)
Supplement: Supplementary file 2 — Reporting Summary [file 41587_2023_2055_MOESM2_ESM.pdf]

nature portfolio

Corresponding author(s): Daniel Pipeleers

Last updated by author(s): Oct 23, 2023

Reporting Summary

Nature Portfolio wishes to improve the reproducibility of the work that we publish. This form provides structure for consistency and transparency in reporting. For further information on Nature Portfolio policies, see our Editorial Policies and the Editorial Policy Checklist.

Statistics

For all statistical analyses, confirm that the following items are present in the figure legend, table legend, main text, or Methods section.

n/a	Confirmed	
☐	☒	The exact sample size (*n*) for each experimental group/condition, given as a discrete number and unit of measurement
☒	☐	A statement on whether measurements were taken from distinct samples or whether the same sample was measured repeatedly
☐	☒	The statistical test(s) used AND whether they are one- or two-sided<br>*Only common tests should be described solely by name; describe more complex techniques in the Methods section.*
☒	☐	A description of all covariates tested
☒	☐	A description of any assumptions or corrections, such as tests of normality and adjustment for multiple comparisons
☐	☒	A full description of the statistical parameters including central tendency (e.g. means) or other basic estimates (e.g. regression coefficient) AND variation (e.g. standard deviation) or associated estimates of uncertainty (e.g. confidence intervals)
☒	☐	For null hypothesis testing, the test statistic (e.g. *F*, *t*, *r*) with confidence intervals, effect sizes, degrees of freedom and *P* value noted<br>*Give P values as exact values whenever suitable.*
☒	☐	For Bayesian analysis, information on the choice of priors and Markov chain Monte Carlo settings
☒	☐	For hierarchical and complex designs, identification of the appropriate level for tests and full reporting of outcomes
☒	☐	Estimates of effect sizes (e.g. Cohen's *d*, Pearson's *r*), indicating how they were calculated

Our web collection on statistics for biologists contains articles on many of the points above.

Software and code

Policy information about availability of computer code

Data collection	Clinical data:<br>Medidata RAVE electronic data capture is the main interface used to collect clinical trial data. Glooko (known as Diasend in the EU) is utilized to capture continuous glucose monitoring data. Data on insulin dose and carbohydrate intake from patients on insulin pump are extracted from Carelink therapy management software. Central laboratory samples are tested and reported by ACM.<br><br>Histology and morphometry data:<br>Axioplan 2 microscope (Carl Zeiss, Oberkochen, Germany) with an Orca-R2 camera (Hamamatsu Photonics, Hamamatsu City, Japan)<br>ImageXpress Pico (Molecular Devices).<br>Aperio CS2 and Aperio GT450 slide scanners, Leica Microsystems Belgium
Data analysis	Clinical data:<br>Glooko software allows for extrapolation of time-in-range data.<br><br>Histology and morphometry data:<br>CellReporterXpress by Molecular Devices Version 2.9.1<br>Iplab Pathway By Becton Dickinson Version 4.06<br>NIS Elements by Nikon version 5.21.01<br>Histoserver, PMA viewer by Pathomation version1.2.1<br>Smartcapture by Digital Scientific version 3.0.8

For manuscripts utilizing custom algorithms or software that are central to the research but not yet described in published literature, software must be made available to editors and reviewers. We strongly encourage code deposition in a community repository (e.g. GitHub). See the Nature Portfolio guidelines for submitting code & software for further information.

Data

Policy information about availability of data

All manuscripts must include a data availability statement. This statement should provide the following information, where applicable:

- Accession codes, unique identifiers, or web links for publicly available datasets
- A description of any restrictions on data availability
- For clinical datasets or third party data, please ensure that the statement adheres to our policy

The tables provide all data that support the statements in the manuscript;
For Table 1 and supplem. Tables 2, 3 and 4, data were derived from the clinical data base for which Dr Manasi Jaiman can be contacted to obtain more information;
For Table 2 and supplem. Table 5, data were derived from a data base held at Vrije Universiteit Brussel where Dr Pipeleers can be contacted for access.

Human research participants

Policy information about studies involving human research participants and Sex and Gender in Research.

Reporting on sex and gender	Recruitment criteria did not make exclusions in terms of sex and gender
Population characteristics	Patients with Type 1 Diabetes Mellitus and Hypoglycemia Unawareness
Recruitment	Patients were recruited based on the established inclusion and exclusion criteria as detailed in the study protocol.
Ethics oversight	UC Davis Clinical Committee A (IRB ID 1525505-3)<br>University of Minnesota Human Research Protection Program (IRB ID 1701M04082)<br>Medical Ethics Committee UZ Brussel/VUB (IRB ID 2020/192)<br>The University of British Columbia Office of Research Ethics - Clinical Research Ethics Board (IRB ID H17-00027)<br>WCG IRB - Office of Human Research Subjects Protection, City of Hope National Medical Center (IRB ID 20172340)

Note that full information on the approval of the study protocol must also be provided in the manuscript.

Field-specific reporting

Please select the one below that is the best fit for your research. If you are not sure, read the appropriate sections before making your selection.

☒ Life sciences ☐ Behavioural & social sciences ☐ Ecological, evolutionary & environmental sciences

For a reference copy of the document with all sections, see nature.com/documents/nr-reporting-summary-flat.pdf

Life sciences study design

All studies must disclose on these points even when the disclosure is negative.

Sample size	This manuscript is an interim report on a cohort of 10 subjects in a Phase 1/2, open-label study on safety, tolerability and efficacy of VC-02 in subjects with type 1 diabetes mellitus and hypoglycemia unawareness (clinicaltrials.gov NCT03163511).
Data exclusions	Data presented in this manuscript are specific to ten patients enrolled in the most recent portion of the clinical trial. Data for patients previously published in references 17 and 18 have not been included.
Replication	The patient case studies presented in the manuscript reflect patients who were enrolled within the same group of the clinical trial. These patients all received the same treatment of device configuration implanted in the same anatomical locations and dose not previously implemented in earlier phases of the clinical study.
Randomization	There is no randomization of patients in this Phase 1/2 clinical trial protocol design. Study entrance criteria require all patients to be C-peptide negative (<0.2 ng/mL) to help establish proof-of-concept increases in C-peptide are product-derived.
Blinding	This clinical trial protocol is designed as open-label since it requires surgical procedures and would be unethical to implant dummy or placebo product without the potential for therapeutic benefit.

Reporting for specific materials, systems and methods

We require information from authors about some types of materials, experimental systems and methods used in many studies. Here, indicate whether each material, system or method listed is relevant to your study. If you are not sure if a list item applies to your research, read the appropriate section before selecting a response.

Materials & experimental systems

n/a	Involved in the study	
☐	☒	Antibodies
☒	☐	Eukaryotic cell lines
☒	☐	Palaeontology and archaeology
☐	☒	Animals and other organisms
☐	☒	Clinical data
☐	☒	Dual use research of concern

Methods

n/a	Involved in the study	
☒	☐	ChIP-seq
☒	☐	Flow cytometry
☒	☐	MRI-based neuroimaging

Antibodies

Antibodies used

1. Guinea pig anti-insulin polyclonal (1/1000, in-house produced).
2. Rabbit anti-glucagon polyclonal (1/1000, in-house produced).
3. Mouse anti-glucagon, monoclonal K79bB10, ascites fluid (Sigma, G2654, lot 084M4793,1/500), RRID:AB_259852.
4. Rat anti-somatostatin, monoclonal M09204 (Abcam, ab30788, 1/100), RRID:AB_778010.
5. Mouse anti-CK19, monoclonal RCK108 (Agilent, M0888, lot 41377336, 1/20), RRID:AB_2234418.
6. Rabbit anti-(wide spectrum) Cytokeratin, polyclonal (Agilent, z0622, lot1015917, 1/200), RRID:AB_2650434.
7. Mouse anti-Chromogranin A, IgG1 mixture of two clones LK2H10 and PHE5 (Thermo Fisher, Ma5-13287, Lot 1015917, 1/500), RRID:AB_10985130.
8. Rabbit anti-CD34, monoclonal [EP373Y] (Abcam, ab81289, Lot GR3240236-15 / GR3240236-11, 1/50), RRID:AB_1640331.
9. Rabbit recombinant monoclonal antiCD4 (SP35), rabbit monoclonal anti-CD8 (SP53), mouse monoclonal antiCD20 (L26), mouse recombinant monoclonal anti CD68 (KP1) all validated in clinical pathology department UZBrussels,using Benchmark Ultra System (Roche Tissue Diagnostics)
10.Donkey anti mouse Alexa Fluor488 (715-546-151, 1/500, Jackson ImmunoResearch Europe), RRID:AB_2340850.
11.Donkey anti mouse Cy3 (715-166-151, 1/500, Jackson ImmunoResearch Europe), RRID:AB_2340817
12.Donkey anti mouse Alexa Fluor 647 (715-606-151, 1/500, Jackson ImmunoResearch Europe), RRID:AB_2340866
13. Donkey anti Rabbit Alexa Fluor 488 (711-546-152, 1/500, Jackson ImmunoResearch Europe), RRID:AB_2340619.
14. Donkey anti Rabbit Cy3 (711-166-152, 1/500, Jackson ImmunoResearch Europe), RRID:AB_2313568.
15. Donkey anti Rabbit Alexa Fluor 647 (711-606-152, 1/500, Jackson ImmunoResearch Europe), RRID:AB_2340625.
16. Donkey anti Guinea pig Alexa Fluor 488 (706-546-148, 1/500, Jackson ImmunoResearch Europe), RRID:AB_2340473.
17. Donkey anti Guinea pig Cy3 (706-166-148, Jackson ImmunoResearch Europe), RRID:AB_2340461.
18. Donkey anti Guinea Pig Alexa Fluor 647 (706-606-148, 1/500, Jackson ImmunoResearch Europe), RRID:AB_2340477.

Validation

1. Guinea pig anti-insulin polyclonal Used for IHC on human pancreas (In't Veld P, Diabetes, PMID: 20413508).
2. Rabbit anti-glucagon polyclonal Used for IHC on human pancreas (In't Veld P, Diabetes, PMID: 20413508).
3. Mouse anti-glucagon,Used for IHC on pancreas tissue (Witt S, Acta Histochem, PMID: 3138717).
4. Rat anti-somatostatin, Used for IHC on human pancreas (Riopel M, Islets. PMID: 25425025).
5. Mouse anti-CK19, Used for IHC on human breast carcinomas (Dalal P, Mod Pathol, PMID:756794).
6. Rabbit anti-(wide spectrum) Cytokeratin, Used for IHC on liver (Frentzas S, Nat Med., PMID: 27748747).
7. Mouse anti-Chromogranin A, Used for human pituitary adenomas (Lu JQ, Endocr Pathol, PMID: 26187094).
8. Rabbit anti-CD34, Used for IHC on Human kidney.

Animals and other research organisms

Policy information about studies involving animals; ARRIVE guidelines recommended for reporting animal research, and Sex and Gender in Research

Laboratory animals	9 - 11 week old Rowett Nude rats
Wild animals	Not applicable.
Reporting on sex	Only male rats were used.
Field-collected samples	Not applicable.
Ethics oversight	Ethical Committee for Animal Experiments - Vrije Universiteit Brussel (15-274-1, 19-274-1)

Note that full information on the approval of the study protocol must also be provided in the manuscript.

Clinical data

Policy information about clinical studies

All manuscripts should comply with the ICMJE guidelines for publication of clinical research and a completed CONSORT checklist must be included with all submissions.

Clinical trial registration	NCT03163511
Study protocol	A summary of the study protocol is provided with this manuscript.

Data collection	Data represented in the manuscript reflect patients enrolled in the trial between August 2020 and October 2021.
Outcomes	The protocol specified the Primary Efficacy Endpoint as change from baseline to week 26 in plasma C-peptide following MMTT, and the following Secondary Efficacy Endpoints for follow-up to maximally 104 weeks:<br>- Change from baseline of C-peptide response to MMTT and percent subjects achieving levels > 0.07 nmol/l,<br>- Change from baseline in average insulin dose and percent subjects with 50 percent reduction, and percent with insulin-independence<br>- Percent of time with blood glucose values <54 mg/dl, 54 to < 70 mg/dl, 70 to ≤ 180mg/dl, > 180 mg/dl (CGM device) and change from baseline in time-in-hypoglycemic range (<70 mg/dl), time-in-euglycemic range (70-180 mg/dl), time-in-hyperglycemic range (>180 mg/dl). Frequency hypoglycemic events-HE and percent subjects free of HE.

Dual use research of concern

Policy information about dual use research of concern

Hazards

Could the accidental, deliberate or reckless misuse of agents or technologies generated in the work, or the application of information presented in the manuscript, pose a threat to:

No	Yes	
☒	☐	Public health
☒	☐	National security
☒	☐	Crops and/or livestock
☒	☐	Ecosystems
☒	☐	Any other significant area

Experiments of concern

Does the work involve any of these experiments of concern:

No	Yes	
☒	☐	Demonstrate how to render a vaccine ineffective
☒	☐	Confer resistance to therapeutically useful antibiotics or antiviral agents
☒	☐	Enhance the virulence of a pathogen or render a nonpathogen virulent
☒	☐	Increase transmissibility of a pathogen
☒	☐	Alter the host range of a pathogen
☒	☐	Enable evasion of diagnostic/detection modalities
☒	☐	Enable the weaponization of a biological agent or toxin
☒	☐	Any other potentially harmful combination of experiments and agents
