# Supplementary material for: Encapsulated stem cell–derived β cells exert glucose control in patients with type 1 diabetes
Source: Nat Biotechnol. 2023 Nov 27;42(10):1507–14. doi: 10.1038/s41587-023-02055-5 (PMC11471599; doi:10.1038/s41587-023-02055-5)
Supplement: Supplementary file 3 — Clinical protocol [file 41587_2023_2055_MOESM3_ESM.pdf]

AN OPEN-LABEL, FIRST-IN-HUMAN, STUDY EVALUATING THE SAFETY, TOLERABILITY, AND EFFICACY OF VC-02™ COMBINATION PRODUCT IN SUBJECTS WITH TYPE 1 DIABETES MELLITUS AND HYPOGLYCEMIA UNAWARENESS

**Protocol Number**	VC02-101
**Compound**	PEC-01™ cells in a Delivery Device (together known as VC-02™ combination product)
**Study Phase**	1/2
**Sponsor Name and Address**	ViaCyte Inc.<br>3550 General Atomics Ct.<br>San Diego, CA 92121
**Version Number**	8.0 (Amendment #9)

Property of ViaCyte, Inc.

PROTOCOL SUMMARY

Title:	An Open-Label, First-in-Human, Study Evaluating the Safety, Tolerability, and Efficacy of VC-02™ Combination Product in Subjects with Type 1 Diabetes Mellitus and Hypoglycemia Unawareness
Phase:	1/2
Population:	Subjects with type 1 diabetes mellitus (T1DM) and hypoglycemia unawareness (HU) will be enrolled into this first-in-human (FIH) clinical trial<br><u>Cohort 1</u>: At least three (3) subjects, but up to 15 total<br><u>Cohort 2</u>: Up to 60 subjects enrolled<br>Total enrollment may be approximately 75 subjects
Number of Sites:	Approximately ten (10)
Study Duration:	The total duration of the trial is estimated to be up to 66 months:<br>• Up to 42 months to complete enrollment of both cohorts<br>• An additional 24 months from the time the last subject is enrolled in Cohort 2 until the last subject last visit
Subject Participation Duration:	Including Screening, Treatment, and Follow-up Visits, each subject's duration of participation is estimated as 110 weeks total (approximately two years).<br>After all VC-02 units have been explanted, each subject will be required to be followed in a separate, long-term, follow-up study.
Description of Agent or Intervention:	ViaCyte has developed the VC-02 combination product, which is intended to control blood glucose in a more physiologic, sensitive, and homeostatic manner than the various forms of injectable insulin and pump therapies currently available. VC-02 combination product is comprised of two distinct components: (1) PEC-01 pancreatic endoderm cells derived from human embryonic stem cells (hESC) and (2) a durable, removable, Delivery Device (DD) designed to deliver and retain cells at the local implant site.<br>Following subcutaneous implantation in anatomical locations involving the trunk or extremities, the VC-02 units are expected to vascularize adequately and the PEC-01 cells are expected to

Property of ViaCyte, Inc.

	differentiate into mature glucose-responsive, insulin-producing cells, capable of secreting insulin in response to serum glucose concentration.<br><br>Subjects may be implanted with VC-02-300 combination product for dose-finding, and smaller VC-02-20 combination product as sentinel units; these are smaller units that will be explanted at various time points and examined ex vivo.<br><br>An immunosuppression regimen will be used to facilitate the engraftment and long-term function of implanted VC-02 units. The overall regimen may vary between subjects but may include the use of basiliximab, anti-thymocyte globulin (ATG), etanercept, sirolimus, tacrolimus, steroids and/or mycophenolate mofetil (MMF). Additional medications can be prescribed by the Investigator to increase the safety and efficacy potential of the product after consultation with the Sponsor.
Study Design:	This will be an open-label, FIH, clinical trial in subjects with T1DM and HU.<br><br>Two cohorts are planned for enrollment in this trial:<br><br><u>Cohort 1 – Initial Safety and Tolerability</u>: Up to 15 subjects may be implanted with up to two (2) VC-02-300 units and up to six (6) VC-02 sentinels at one (1) or more clinical sites. If no VC-02-300 units are implanted in a subject, up to ten (10) VC-02 sentinels may instead be implanted. Sentinel units may be explanted at varying time points post-implant to assess the status of cell viability and differentiation, vascularization, and host response. At a minimum, the first three (3) subjects in Cohort 1 will be enrolled sequentially in order to assess safety and tolerability data at Week 2 before implanting the next subject. If no serious, treatment-related adverse events (AEs) are observed with the first three (3) subjects after each has reached Week 2, subsequent Cohort 1 enrollment may be performed in parallel. Total duration of treatment (implantation) may be up to two (2) years for each Cohort 1 subject, with the last unit explanted at Month 24. Cohort 1 subjects will complete a total of 18 study visits.<br><br>After a minimum of three (3) subjects have been enrolled in Cohort 1 and have completed thru Week 4, the Data Safety Monitoring Board (DSMB) will review the cumulative Cohort 1 data for safety, tolerability, and proof of mechanism.<br><br><u>Cohort 2</u> – Up to 60 subjects will be implanted in cadres and will test a particular device configuration and/or implant strategy.

Property of ViaCyte, Inc.

Based on information obtained from subject explants within a given cadre, the need for an alternate device configuration and/or surgical implant technique (e.g., pharmacological intervention, implant site, etc.) may be identified in order to improve engraftment and cell survival outcomes. These changes will be implemented prior to the next cadre of subjects commencing implantation to drive VC-02 engraftment optimization.

Cohort 2 subjects will be implanted with up to twelve (12) units. Of the twelve implanted units, no more than ten (10) will be VC-02-300 and the remainder will be VC-02-20 units. For example, if ten (10) VC-02-300 units are implanted in a subject, two (2) VC-02 sentinels may be implanted. Cohort 2 enrollment will be competitive across approximately ten (10) sites. Sentinel units may also be explanted at various time points post-implant.

Total duration of treatment may be up to two (2) years for each Cohort 2 subject, with the last unit explanted at Month 24. Cohort 2 subjects will complete a total of up to 18 study visits.

Study Objectives:

Objectives: This trial will test whether VC-02 combination product can be implanted and maintained with safety, tolerability and efficacy for up to two years. There are two cohorts in this FIH trial with the following study objectives.

Cohort 1 study objectives:

- Assess the local and systemic safety and tolerability of VC-02 combination product when implanted into subjects with T1DM and HU.
- Assess histological proof of mechanism for VC-02 combination product (e.g., cell survival and differentiation to beta cells).

Cohort 2 study objectives:

- Evaluate the clinical efficacy and further assess safety and tolerability of VC-02 combination product from implantation to Month 24.
- Explore effects of weight, gender, BMI, or other potentially interacting factors on the responsiveness of the subjects to the experimental intervention.

Exploratory objectives:

- Optimize the recommended surgical implantation procedure, anatomical location, and perioperative care for VC-02.

Property of ViaCyte, Inc.

- Assess the effects of the host immune response to implanted VC-02 units

Primary Endpoints: The primary endpoints vary between the two (2) cohorts and include safety, tolerability, and efficacy.

- Cohort 1: Targeted safety and tolerability profile inclusive of:
  - The incidence of AEs with causality related to VC-02 combination product, the surgical procedures required for VC-02 administration, and the immunosuppressive drug regimen.
  - The incidence of off-target growth as evidenced by implanted VC-02 units via lumen ultrasound measurements, or by histological examination of explants.
  - The incidence of immune sensitization defined by presence of donor anti-HLA antibodies absent prior to implant.
  - Implant tolerability assessments (e.g., fever, erythema, pain, tenderness, induration) for up to four hours post-implantation and at subsequent visits.
- Cohort 2: Change from baseline to Week 26 in C-peptide $AUC_{0\text{-}4h}$ following a Mixed Meal Tolerance Test (MMTT)

Secondary Endpoints: Safety and Tolerability: Comprehensive profile of VC-02 combination product implanted for up to two years as measured by:

- All reported AEs
- The incidence of immune sensitization defined by presence of donor anti-HLA antibodies absent prior to implant
- Implant tolerability assessments (e.g., fever, erythema, pain, tenderness, induration) for up to four hours post-implantation and at subsequent visits
- The incidence of subjects requiring a premature explant due to safety, tolerability, or malfunction issues

Efficacy:

- Change from baseline to Weeks 16, 26, 39, 52, 78, and 104 in average daily insulin dose in the seven days preceding the Clinic Visit

Property of ViaCyte, Inc.

- Percent of subjects who achieve a 50% reduction in average weekly exogenous insulin dose from baseline to Weeks 16, 20, 26, 39, 52, 78, and 104
- Percent of subjects who achieve exogenous insulin independence; of those subjects achieving insulin independence, the percent achieving HbA1c <7.0%
- Percent of time spent with blood glucose values at various cut points (e.g., <54 mg/dL, ≥54 to <70 mg/dL, ≥70 mg/dL to ≤180 mg/dL, and >180 mg/dL) as measured by each subject's continuous glucose monitoring (CGM) device
- Change from baseline to Weeks 16, 26, 39, 52, 78, and 104 in time-in-euglycemic range (≥70 mg/dL to ≤180 mg/dL), time-in-hypoglycemic ranges (<54 mg/dL and ≥54 to <70 mg/dL), and time-in-hyperglycemic ranges (>180 mg/dL) as measured by each subject's CGM

Exploratory Endpoint:

Histological results of explanted units and any associated tissue capsule as evaluated for cell viability, vascularization, immune response, and/or cell maturation and differentiation.

Inclusion Criteria:

- Signed and dated informed consent form
- Men and non-pregnant women of 18-65 years of age
- Diagnosis of T1DM for a minimum of five (5) years
- At least one severe hypoglycemic event, or for patients with CGMs, documentation of a low blood glucose value <54 mg/dL (<3.0 mmol/L), in the previous 12 months
- Documented hypoglycemia unawareness (Clarke score ≥4) or significant glycemic lability as assessed by the Investigator
- Stable, optimized diabetic regimen for at least 3 months
- Insulin dosage at screening <1 unit/kg/day
- Willing to use a provided CGM System
- Willing and able to comply with daily entries on a study diary
- All male subjects and female subjects of childbearing potential must practice effective contraception during the study and be willing and able to continue contraception until final explant. For further details of contraceptive requirements for this study, please refer to Section 5.4.5

Property of ViaCyte, Inc.

Exclusion Criteria:	• A detectable stimulated serum C-peptide at any time-point during the Screening period, defined as >0.2 ng/mL (>0.0667 nmol/L)<br>• Use of any oral diabetes-specific medication<br>• Medical history of islet cell, kidney, and/or pancreas transplant<br>• Occurrence of six or more severe, unexplained hypoglycemic events within six months of enrollment<br>• Uncontrolled or untreated thyroid disease or adrenal insufficiency<br>• Known causes of diabetes other than T1DM<br>• Diabetic complications such as severe kidney disease or renal dysfunction, proliferative retinopathy, diabetic foot ulcers, amputations attributable to diabetes, and/or severe peripheral neuropathy<br>• Non-compliance with current anti-diabetic regimen<br>• Hemoglobin A1C level of ≥10.0%<br>• Significant skin conditions involving the area(s) targeted for implantation<br>• Alcohol abuse<br>• Positive urine drug screen for substances of abuse at screening or enrollment visit, medical marijuana use may be allowed by the PI after consultation with the Medical Monitor and/or Sponsor<br>• Prior history of malignancy with the exception of:<br>o Basal cell carcinoma of the skin;<br>o Squamous cell carcinoma of the skin that has been recurrence free for ≥five years;<br>o Appropriately treated in situ carcinoma of the cervix<br>• Known allergies to portions of the cellular excipients used as cell preservation solution or the PEC-01 manufacturing process (i.e., bovine, porcine allergies)<br>• History of severe asthma or COPD<br>• BMI ≥32 kg/m2 or <18 kg/m2 at screening

Property of ViaCyte, Inc.

- Active hepatobiliary disease or an AST or ALT >1.5 x ULN at screening or a total bilirubin >1.5 x ULN unless the subject has a history of Gilbert's disease
- Active infection or known history of Hepatitis B or C or HIV
- Evidence of previous TB infection (including BCG vaccination or positive PPD)
- Negative serostatus for Epstein-Barr virus
- Other abnormal labs at screening:
    - Platelets <100,000
    - Hgb <12 g/dL (males) or <11 g/dL (females)
    - Fasting triglycerides >500 mg/dL
    - Estimated Glomerular Filtration Rate (GFR) <60 mL/min/1.73 m2 (using MDRD calculator)
- Clinical lab value outside normal range, unless deemed as not clinically significant by the Investigator and Sponsor
- Sustained hypertension defined as average systolic ≥160 mmHg or diastolic ≥90 mmHg at screening
- 12-lead ECG findings demonstrating:
    - QTc>450 msec for males or >470 msec for females at screening
    - Any other abnormality deemed clinically significant requiring further clinical evaluation by the Investigator
- Any history of unstable angina or Class 3 or 4 CHF, or any of the following diagnoses/conditions or procedures within the past year: stroke, myocardial infarction, life-threatening arrhythmia, major cardiovascular procedure (e.g., angioplasty, planned angioplasty, or carotid endarterectomy), or any other clinically significant cardiovascular disease diagnosis or procedure
- History of coagulopathy
- Participation in a study of an investigational drug, device, or graft within five half-lives of the experimental agent or 30 days prior to enrollment in this study, whichever is longer
- Planned surgery in the general location of the implanted units (i.e., back and/or flank, arms, legs, abdomen, etc.) at any time during study participation

Property of ViaCyte, Inc.

Statistical Considerations	Sample Sizes – Cohorts 1 and Cohort 2<br>• Sample size in Cohort 1 was empirically derived, based upon safety considerations and data accumulated from previously performed ViaCyte clinical trials. A sample size of up to 15 subjects should allow for adequate assessment of Cohort 1 study objectives.<br>• A sample size of 60 subjects in Cohort 2 will enable staggered enrollment of cadres of subjects allowing for testing of alternate device configurations and/or surgical implant technique (e.g., pharmacological intervention, implant site, etc.) in order to improve engraftment and cell survival outcomes. This approach will drive VC-02 engraftment optimization.<br>Sample Size – Primary Efficacy Endpoint<br>• Once the optimized device configuration and surgical implant technique is determined, a sample size of 40 subjects implanted under those conditions will provide >80% power to detect a mean change from baseline to Week 26 in C-peptide AUC_{0-4h} of 3.0 ng x hour/mL following an MMTT, assuming a standard deviation of 6.0. Other assumptions include a two-sided test with $p \geq 0.05$.<br>If the minimal level of detection for C-peptide is 0.20 ng/mL, this would give an AUC of 0.8 ng x hour/mL for the 4-hour MMTT. A sample size of 40 subjects provides >80% power to detect a change from baseline to Week 26 in C-peptide AUC_{0-4h} of 0.8 ng x hour/mL following an MMTT, assuming a standard deviation of 1.6.<br>For the 2-hour MMTT, a sample size of 40 subjects provides >80% power to detect a change from baseline to Week 26 in C-peptide AUC_{0-2h} of 0.4 ng x hour/mL following a MMTT (if the minimal level of detection for C-peptide is 0.20 ng/mL), assuming a standard deviation of 0.8.<br>Analysis Populations<br>• The Full Analysis Set (FAS) is defined as Cohort 2 subjects enrolled into the study and who received implantation of at least one VC-02 unit on Study Day 1. All efficacy summaries/analyses will be performed on the FAS. Subjects will be summarized by treatment group.<br>• The Safety Analysis Set (SAS) will include all Cohort 1 and Cohort 2 subjects enrolled into the study and in whom

Property of ViaCyte, Inc.

an implant surgery was attempted, regardless if any VC-02 units were actually implanted.

Primary Safety

- The SAS will be used for the primary safety summarizations. Adverse events and SAEs will be summarized by system organ class (SOC), by severity, and by relationship. This will be done by treatment group and overall.
- The summarization of AEs will focus on only those events that are TEAEs, but the AE listings will include all reported AEs regardless of when they started.
- Other safety data, such as vital signs and clinical laboratory data will be summarized by study visit and treatment group. Where appropriate, change from baseline in safety data will also be summarized in a similar manner.
- The number of subjects undergoing a premature VC-02-300 unit explant will be provided in a listing which includes the reason for explantation.

Primary Efficacy

- Change from baseline to Week 26 in C-peptide $AUC_{0\text{-}4h}$ following an MMTT will be analyzed using analysis of covariance (ANCOVA), with treatment group as a factor and baseline C-peptide $AUC_{0\text{-}4h}$ as a covariate. The FAS will be used to analyze the primary efficacy endpoint. The output from the ANCOVA will include the least squares mean (LSM) and standard error (SE) for each treatment group.

Property of ViaCyte, Inc.
